# Skinspan: A Holistic Roadmap for Extending Skin Longevity With Evidence‐Based Interventions

**DOI:** 10.1111/jocd.70432

**Published:** 2025-09-06

**Authors:** Elizabeth Kream, Sabrina G. Fabi, Monica Boen

**Affiliations:** ^1^ Cosmetic Laser Dermatology San Diego California USA

## Abstract

**Background:**

With the rise of regenerative medicine and geroscience, translational research has shifted focus from lifespan to healthspan—years lived in good health. Applied to aesthetic medicine, the authors introduce the concept of “skinspan,” to both describe the period during which skin maintains a youthful, healthy appearance, and additionally to serve as a tool for the cosmetic consult.

**Aims:**

The aim of this comprehensive review is to illuminate “skinspan” as a framework for guiding long‐term skin health. The authors review the molecular drivers of skin aging along with appraise current evidence‐based interventions and synthesize evidence into an algorithm to expand skin span.

**Methods:**

A comprehensive literature review was conducted to examine the molecular hallmarks of skin aging: genomic instability, mitochondrial dysfunction, cellular senescence, and proteostasis decline, and how they are targeted by lifestyle modifications, pharmacologic agents, and aesthetic procedures. Each intervention discussed in this paper is categorized based on the strength of the available evidence for augmenting skinspan.

**Results:**

Proactive interventions, including lifestyle interventions, topical agents, systemic therapies, and noninvasive procedures, show promise in mitigating aging mechanisms and preserving skin health. We recommend a first‐line strategy of sun protection, topical retinoids, and antioxidants. Second‐line interventions include laser and energy‐based devices. Clinicians may also consider emerging therapies—including stem cell–based treatments, sirtuins, nicotinamide, and natural SIRT activators—as adjunctive third‐line add‐ons, while continuing to monitor the evolving evidence base.

**Conclusion:**

The skinspan framework offers a holistic, evidence‐based approach to skin longevity, promoting prevention, continuity of care, and patient education beyond isolated aesthetic interventions. To expand skinspan based on current evidence, we recommend a first‐line approach of sun protection, topical retinoids, and antioxidants. Second‐line interventions include procedures such as laser and energy‐based devices. Clinicians may also consider emerging therapies—including stem cell–based treatments, sirtuins, nicotinamide, and natural SIRT activators—as adjunctive third‐line add‐ons, while continuing to monitor the evolving evidence base. A need exists for more randomized controlled trial studies to strengthen the evidence base.

## 1 Introduction

As the body's largest organ, the skin serves not only as a protective barrier against environmental insults but also as a visible indicator of health, vitality, and age. Skin aging presents with phenotypic changes such as rhytides, dyspigmentation, laxity, thinning, and increased vulnerability to neoplasms. These phenotypic skin changes are underpinned by an interplay of intrinsic and extrinsic factors, all of which converge on shared molecular pathways that disrupt skin homeostasis over time.

**FIGURE 1:**
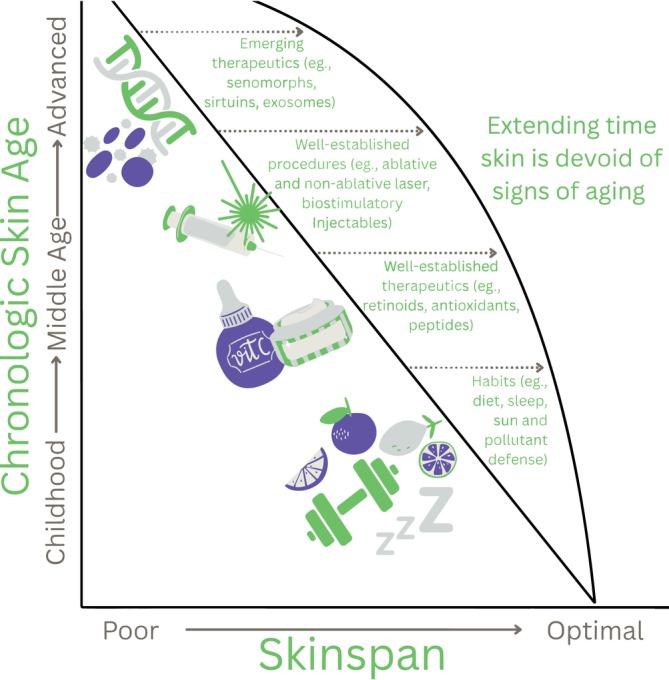
Skinspan refers to the preservation of skin health and appearance relative to chronological age, which may be optimized through lifestyle, medical, and procedural factors.

Establishing the molecular drivers of aging has led to a shift in medicine from prioritizing lifespan, the total years lived, to healthspan, defined as the number of years lived in good health and free of significant morbidity. Building on this paradigm, the concept of skinspan describes the period of youthful, healthy skin, with Figure 1 illustrating how interventions may prolong it. While the term ‘skinspan’ has been coined in informal contexts, to our knowledge, this publication represents the first instance of the term being introduced in peer‐reviewed literature, accompanied by recommendations for its integration into clinical practice. This paper reviews core molecular mechanisms of skin aging along with how these hallmarks are targeted through proactive interventions, altogether extending skinspan.

## 2 Molecular Hallmarks of Skin Aging

The molecular signatures of skin aging unfold in a progressive sequence: damage is induced at the cellular level, cells respond to this damage through adaptive or maladaptive mechanisms, and eventually these responses culminate in a senescent, dysfunctional tissue phenotype.

### 2.1 Damage Accumulates: Genomic Instability, Telomere Attrition, and Loss of Proteostasis

The initial stage of skin aging, on a molecular level, is heralded by damage induction. Skin cells face constant assaults from ultraviolet radiation (UVR), pollution, metabolic byproducts, and internal replication errors. These stressors lead to point mutations, strand breaks, and mitochondrial DNA damage, all of which contribute to the deterioration of cellular function [1]. Telomere attrition, driven by decreased telomerase activity, leads to a loss of chromosomal protection and triggers cellular senescence [1]. Simultaneously, the decline of proteostasis results in the accumulation of damaged proteins and advanced glycation end products (AGEs), which stiffen the dermal matrix [1, 2]. This sets the stage for progressive cellular dysfunction.

### 2.2 Damage Control Declines: Mitochondrial Exhaustion and Cellular Senescence

Compensatory mechanisms designed to defend against damage become overwhelmed and defective with age. Defective mitochondrial polymerase leads to a premature aging phenotype seen in murine models [3]. Senescent fibroblasts reduce their production of insulin‐like growth factor 1 (IGF‐1), a hormone that signals keratinocytes to repair from UV‐induced DNA damage [4]. Consequently, it can be generally stated that changes in the aged skin occur in the dermis and between the epidermis and the dermis.

### 2.3 Senescent Skin: The Rise of “Zombie Cells” and Inflammaging

Senescent cells adopt a pro‐inflammatory secretory profile known as the senescence‐associated secretory phenotype (SASP), characterized by cytokines such as interleukin‐6 (IL‐6), interleukin‐1β (IL‐1β), and tumor necrosis factor alpha (TNF‐α) [5]. The SASP induces senescence in neighboring cells—a domino effect that has led to the term “zombie cells” being ascribed to senescent cells [5]. In the skin, an increase in senescent cells, such as melanocytes, is associated with wrinkling and higher perceived age [6]. Tissue enters an “inflammaging” state, observed in aging skin but also in age‐related diseases such as cancer, atherosclerosis, and diabetes [5].

## 3 Lifestyle Factors That Support Skinspan

### 3.1 Photoprotection

UVR is the main extrinsic driver of skin aging, responsible for 80% to 90% of facial aging [7, 8]. UVB causes epidermal DNA damage and sunburn, while deeper‐penetrating UVA has deleterious effects on fibroblast gene expression [9]. Shorter wavelengths (410–420 nm) are also toxic to fibroblasts and promote aberrant melanogenesis via reactive oxygen species (ROS) generation, transforming growth factor beta (TGF‐β), and matrix metalloproteinase‐1 (MMP‐1) upregulation [10, 11, 12].

**FIGURE 2:**
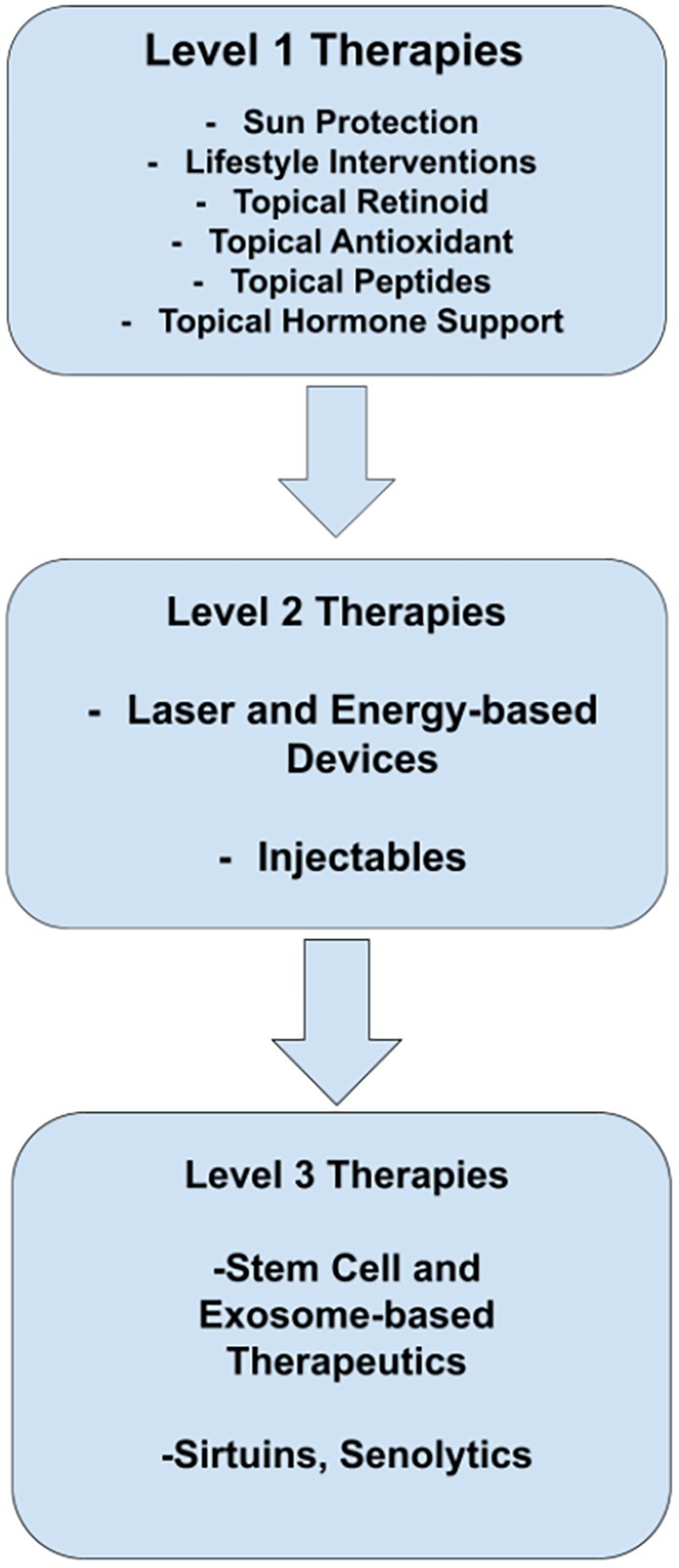
Based on current evidence, we propose this algorithm as a practical tool for clinicians to use during the cosmetic consultation.

In addition to protective clothing and avoiding peak hours of sun exposure, sunscreens suppress UVA‐induced oxidative and inflammatory gene expression in keratinocytes and fibroblasts, with broad‐spectrum UVA/UVB filters offering better protection across all skin phototypes than UVB‐only formulations [13]. Photoprotection is a well‐established first‐line intervention; however, clinicians may find it challenging to stratify second‐ and third‐line recommendations. The authors synthesize the published literature into an algorithm (Figure 2) that encompasses first‐line therapies through emerging interventions, their appraisal of which ensues.

### 3.2 Smoking Avoidance and Cessation

Cigarette smoke accelerates skin aging by inducing oxidative stress, impairing fibroblast function, and disrupting autophagy [14]. Carcinogens in the smoke exhibit phototoxic properties in vitro [15]. While avoidance is ideal, quitting smoking at any age has been associated with improved skin health and reversal of biological skin aging markers [16].

### 3.3 Pollution Control

Airborne pollutants, such as particulate matter (PM), gases, and volatile organic compounds (VOCs), contribute to DNA damage, lipid peroxidation, and impaired autophagy in skin cells [17]. Epidemiologic and mechanistic studies link pollution to pigmentary changes and wrinkle formation [18, 19, 20]. One study introduced the term “environment‐induced lentigines”, Highlighting the aryl hydrocarbon receptor (AHR) signaling pathway's role in pollution and UV‐induced pigmentation via keratinocyte–melanocyte–fibroblast crosstalk [18].

Mitigation strategies include daily cleansing, avoiding outdoor activity during peak pollution, and reducing indoor VOCs by repairing leaks and using low‐VOC products. Because pollution and UVR have compounding effects, sun protection is paramount. Antioxidant‐containing topicals and potential AHR modulators may reduce pollution‐induced inflammation and the SASP production [20, 21].

### 3.4 Diet

Caloric restriction (CR) is one of the most studied lifestyle interventions to extend healthspan; in animal models, CR has been associated with decreased collagen glycation [22], enhanced hair follicle stem cell activity [23], and reduced wrinkle formation [24]. It is important to note that glucagon‐like peptide‐1 receptor agonists (GLP‐1) treatment, increasingly popular for weight loss and weight management, and associated with CR, have been associated with accelerated facial aging due to loss of dermal and subcutaneous fat, altered adipose‐derived stem cell (ADSC) proliferation, and hormonal influences [25, 26].

Limiting dietary glucose and galactose, along with avoiding cooking methods like grilling, frying, and roasting, reduces AGE formation, while steaming and boiling are associated with reduced levels [27].

Antioxidant‐rich diets, such as the Mediterranean diet, lower C‐reactive protein and TNF‐α, and support a diverse gut microbiome—both of which contribute to improved skin and overall health [28, 29]. Among dietary antioxidants, polyphenols, found in fruits, vegetables, dark chocolate, green tea, and spices, confer ROS scavenging and anti‐glycation properties [25, 30].

### 3.5 Sleep

Sleep plays a critical role in cellular repair, DNA restoration, and mitochondrial recovery. Circadian disruption impairs hair cycling and weakens the skin barrier [31]. A study in adults aged 69 to 86 found that sleep deprivation elevated molecular markers of skin aging, including the SASP [32]. In mice, melatonin supplementation restored skin health by normalizing circadian rhythms in the gut microbiome, increasing propionic acid, a key mediator of the gut–skin axis [33].

### 3.6 Exercise

Skeletal muscle functions as an endocrine organ, secreting “myokines.” One myokine, IL‐15, may play a role in preserving skin integrity; mice lacking muscle mass show skin deterioration that can be reversed with recombinant IL‐15 [34].

Exercise also reduces pro‐inflammatory cytokines and increases protective factors like IGF‐1 [35]. A 16‐week study of middle‐aged women found both aerobic and resistance training improved extracellular matrix‐related gene expression, elasticity, and dermal thickness [36]. Regular physical activity also reduced stratum corneum thinning and increased regulators of mitochondrial biogenesis [34].

### 3.7 Mainstay Medical Interventions: Retinoids and Vitamin C

Topical retinoids promote collagen synthesis and epidermal renewal by activating nuclear receptors that regulate gene expression involved in skin structure and repair. Retinoic acid, the active form of vitamin A, is more potent than its precursor retinol, which requires metabolic conversion before receptor activation. Consistent use decreases MMPs and increases collagen type I (COL1) production. In a vehicle‐controlled human study, tretinoin 0.1% cream led to an 80% increase in COL1 formation in photodamaged skin, exhibiting a restorative potential [37].

Vitamin C, particularly in its most studied form, L‐ascorbic acid, enhances skin health through multiple mechanisms: ROS scavenging, tyrosinase inhibition to reduce hyperpigmentation, genomic safeguarding through methylation reversal, and serving as a cofactor for collagen‐stabilizing enzymes [38]. Topical application is more effective than oral supplementation, but formulation with stabilizing carriers is essential. In clinical studies, topical ascorbic acid reduced UVA/UVB‐induced TNF‐α and IL‐1β expression [39] and showed a synergistic benefit when combined with sunscreen in suppressing MMP‐1 expression [40]. Vitamin C also mitigates oxidative skin damage from ozone exposure [41].

### 3.8 Sex Hormones

Hormonal changes associated with menopause and andropause significantly affect skin physiology, contributing to dryness, reduced elasticity, and dermal thinning. Two randomized controlled trials (RTCs) found that 24 weeks of topical estradiol application increased fibroblast numbers and boosted COL1 and COL3 [42, 43]. However, a shorter study with a 30‐day duration did not observe significant effects, suggesting duration and formulation may influence outcomes [44, 45].

Emerging evidence supports the role of oxytocin (OT) in modulating skin aging, particularly in women. A recent in vitro study investigated the effects of OT on human dermal fibroblasts derived from women of different ages [44, 45]. OT suppressed SASP‐induced senescence, and this effect correlated with the hypermethylation of the OT receptor gene in older donors [44, 45]. A double‐blinded RTC of 40 women evaluated a topical formulation of peptides and botanical actives exhibiting OT and pheromone‐like effects, applied over a 4 to 8‐week period [46]. The active group demonstrated a significant 3‐year reduction in perceived skin age [46].

### 3.9 Melatonin

Melatonin, a hormone best known for regulating circadian rhythms, activates sirtuins, inhibits toll‐like receptor 4 signaling, and reduces pro‐inflammatory cytokines associated with inflammaging [47, 48, 49]. Both topical and oral melatonin supplementation resulted in an improvement in androgenetic alopecia [50]. Studies show melatonin's role in downregulating MMP‐1 and the mechanistic target of rapamycin (mTOR) signaling pathway, thereby restoring dermal collagen and elastic fibers [51]. mTOR is a nutrient‐sensing pathway critical for cell growth. When mTOR is overactive, it promotes the accumulation of misfolded proteins and impairs autophagy. Additionally, melatonin protects melanocytes from UVB‐induced pigmentation [52].

### 3.10 Peptides

Bioactive peptides are short amino acid sequences that modulate molecular pathways tied to skin structure and repair. Categories include carrier peptides (e.g., GHK‐Cu), signaling peptides (e.g., tripeptide‐29, matrixyl), enzyme inhibitors, and neurotransmitter‐affecting peptides. Advances in computational screening have accelerated peptide discovery for skin applications. In vitro, ex vivo, and human clinical trials found that the carrier peptide GHK‐Cu, in particular, promotes collagen synthesis, reduces MMPs, and restores expression of stem cell markers such as integrins and p63 [53, 54, 55]. A topical nonapeptide prevented UVA‐induced photoaging in mouse models by suppressing oxidative stress, preserving mitochondrial function, and maintaining autophagy [56].

### 3.11 Stem Cells and Exosome‐Based Therapeutics

Stem cell–based therapies, particularly ADSCs and mesenchymal stem cells, exert their beneficial effects on skin aging through paracrine signaling—secreting cytokines, growth factors, and matrix‐remodeling enzymes that enhance fibroblast function and dermal architecture. ADSCs have been shown in vitro and in vivo to reduce AGEs, modulate UVB‐induced stress pathways, and upregulate collagen synthesis [56, 57]. Clinical studies using autologous lipoaspirates rich in ADSCs showed improvements in skin texture and wrinkle depth after intradermal injection [58]. Plant stem cells, while structurally distinct, have demonstrated regenerative potential in in vivo and clinical trials of topical formulations by mitigating oxidative stress, inflammaging, and fibroblast senescence [59, 60].

Exosomes, nano‐sized extracellular vesicles secreted by all cells, including stem cells, are gaining traction for their ability to deliver bioactive molecules without the complexities of live cell therapy. In vitro and in vivo studies of a topical formulation of exosomes from human umbilical vein endothelial cells and hypoxia‐induced ADSCs enhanced fibroblast proliferation, increased COL1 and COL3 levels, and reduced MMP‐1 expression [61, 62]. In the study utilizing hypoxia conditions, this enhances the natural niche for stem cells and therefore increases exosome yield.

Initial human trials, primarily investigating topical formulations of exosome applied after procedures, demonstrate efficacy in treating skin aging, scarring, melasma, and hair loss, as well as showing potential in inflammatory and pigmentary skin diseases [63]. A 12‐week clinical trial with human participants found topical application of platelet‐derived exosomes reduced markers of cellular senescence and telomere damage [64]. Another in vitro study investigating fibroblast‐conditioned media developed under hypoxic conditions found an upregulation of autophagy and stem cell genes, with correlating clinical improvements noted in collagen and elastin content, wrinkles, sagging, and photodamage [65].

### 3.12 Sirtuins, NAD+, and Natural SIRT Activators

Sirtuins (SIRTs) are oxidized nicotinamide adenine dinucleotide (NAD^+^)‐dependent deacetylases central to cellular longevity, DNA repair, and apoptosis regulation. SIRT1 and SIRT6 are particularly relevant to skin aging. Because SIRT activity depends on NAD^+^, age‐related declines in NAD^+^ impair these protective pathways. Nicotinamide supplementation has been shown to extend fibroblast lifespan, enhance collagen production, and reduce ROS in vitro and ex vivo studies [66, 67].

A 2023 review of preclinical and clinical evidence on NAD^+^ highlights promising early clinical evidence, including reduced risk of nonmelanotic skin cancers and precancers with oral nicotinamide; however, concerns remain that elevated NAD^+^ levels may support cancer cell metabolism via enhanced glycolysis and potentially upregulate the SASP [68]. While oral NAD^+^ precursors lack clear evidence for improving skin aging endpoints, a study of a topical nicotinic acid derivative applied over 4 to 6 weeks increased skin NAD levels and improved multiple markers in photodamaged skin [69].

Natural SIRT activators such as resveratrol, honokiol, lycopene, and cordyceps sinensis extract have all displayed skin antiaging effects through increasing sirtuin expression and NAD+ synthesis in several in vitro and clinical trials [70, 71, 72, 73].

### 3.13 Senotherapeutics

Senolytics eliminate senescent cells via apoptosis, while senomorphs suppress the SASP and support tissue homeostasis. Dasatinib, a senolytic, has shown to selectively clear senescent fibroblasts and improve fibrosis in preclinical skin models [70]. Rapamycin (sirolimus), an mTOR inhibitor with senomorphic properties, has demonstrated an ability to reduce senescent markers and increase markers of dermal youthfulness, with supporting evidence from in vitro and ex vivo studies, and most notably from the first clinical trial evaluating topical rapamycin in photodamaged human subjects [74, 75, 76]. Another senomorph, metformin, was found to promote fibroblast survival and hair regeneration in both in vitro and in vivo models [77, 78, 79].

### 3.14 Nonsurgical Aesthetic Intervention: Lasers, Light‐Based Therapies, and Injectables

Minimally invasive procedures, especially when combined, are foundational in a skin aging regimen. Intense pulsed light enhances fibroblast activity and boosts pro‐COL1/3 and elastin production via photothermal and cytokine‐triggered mechanisms [80]. Fractionated CO₂ laser has been shown to upregulate TGF‐β, heat shock proteins, and COL1 while suppressing MMP‐13 expression, improving both photoaging and skin carcinogenesis markers in preclinical and clinical studies [81, 82]. 1064 nm laser activates rejuvenation pathways in both fibroblasts and keratinocytes, increasing heat shock proteins, procollagens, and TIMPs, while reducing aging‐associated markers [83]. Radiofrequency microneedling induces TGF‐β expression and dermal remodeling proteins including tropoelastin and fibrillin. Poly‐L‐lactic acid, a biostimulatory filler, activates the TGF‐β/Smad pathway to promote procollagen and elastin production [8].

### 3.15 Photobiomodulation (PBM)

PBM utilizes red (590–660 nm) and near‐infrared light, enhances mitochondrial cytochrome c oxidase activity to promote cellular repair, similar to photosynthesis in plants. While concerns exist about whether PBM is a friend or foe in the context of skin aging, exposure to appropriate doses may offer photoprotection against harmful UV radiation, potentially preconditioning the skin [84]. In a split‐face RCT involving 137 women, 10 sessions of red and amber PBM over 4 weeks led to visible improvements in periocular wrinkle depth and skin texture [85]. A study investigating infrared radiation, involving both in vitro and human in vivo groups, found a significant increase in collagen and elastin production proportional to the duration of irradiation exposure [86]. Proteomic data analysis of skin response to PBM revealed increased collagen and elastin levels, and reduced MMPs, indicating activation of fibroblasts and keratinocytes [87].

## 4 Conclusion

We coin the concept of skinspan to serve as a framework for extending the period of youthful skin, and with emerging interventions, potentially reverse signs of aging. The term and Figures can be recruited as a concrete tool during the cosmetic consultation to clearly stratify interventions for patients. In contrast to fragmented reactive treatments leading to perception drift, the skinspan model serves as a skin aging roadmap, steeped in evidence‐based therapies and a trusted relationship between the unique patient and their trusted clinician. Our algorithm, illustrated in Figure 2, outlines a practical approach to expanding skinspan based on current evidence. We recommend a first‐line strategy of sun protection, topical retinoids, and antioxidants. Second‐line interventions include laser and energy‐based devices. Clinicians may also consider emerging therapies—including stem cell‐based treatments, sirtuins, nicotinamide, and natural SIRT activators—as adjunctive third‐line add‐ons, while continuing to keep a pulse on the evolving evidence base. Skinspan also illuminates the synergism of procedural, medical, and lifestyle interventions, many of which confer benefits beyond the skin. Building on the canon of in vitro and in vivo studies, more randomized clinical trials are needed to strengthen the evidence base, especially as new therapeutic candidates continue to emerge.

## Disclosure

The authors have nothing to report.

## Conflicts of Interest

Elizabeth Kream and Monica Boen: The authors declare no conflicts of interest. Sabrina G. Fabi, M, serves as a consultant for AbbVie, Galderma, Merz, and Revance, and receives research funding or serves as an investigator for AbbVie, Caliway, Galderma, Merz, Teoxane, Tigermed, and Symatase. She is also the co‐founder of XOMD skincare. These roles did not influence the design, writing, or interpretation of this manuscript.

## Data Availability

The data that support the findings of this study are openly available in PubMed at https://pubmed.ncbi.nlm.nih.gov/.
